# The function of *Scox* in glial cells is essential for locomotive ability in *Drosophila*

**DOI:** 10.1038/s41598-021-00663-2

**Published:** 2021-10-27

**Authors:** Ryosuke Kowada, Atsushi Kodani, Hiroyuki Ida, Masamitsu Yamaguchi, Im-Soon Lee, Yasushi Okada, Hideki Yoshida

**Affiliations:** 1 Department of Applied Biology, Kyoto Institute of Technology, Matsugasaki, Sakyo-ku, Kyoto, 606-8585 Japan; 2 Advanced Insect Research Promotion Center, Kyoto Institute of Technology, Matsugasaki, Sakyo-ku, Kyoto, 606-8585 Japan; 3 Kansai Gakken Laboratory, Kankyo Eisei Yakuhin Co. Ltd., Seika-cho, Kyoto, 619-0237 Japan; 4 Department of Biological Sciences, Konkuk University, Seoul, Republic of Korea; 5 Laboratory for Cell Polarity Regulation, Center for Biosystems Dynamics Research (BDR), RIKEN, Suita, Osaka 565-0874 Japan; 6 Department of Physics and Universal Biology Institute (UBI), Graduate School of Science, and International Research Center for Neurointelligence (WPI-IRCN), The University of Tokyo, 7-3-1 Hongo, Bunkyo-ku, Tokyo, 113-0033 Japan

**Keywords:** Disease model, Glial biology

## Abstract

*Synthesis of cytochrome c oxidase* (*Scox*) is a *Drosophila* homolog of human *SCO2* encoding a metallochaperone that transports copper to cytochrome c, and is an essential protein for the assembly of cytochrome c oxidase in the mitochondrial respiratory chain complex. *SCO2* is highly conserved in a wide variety of species across prokaryotes and eukaryotes, and mutations in *SCO2* are known to cause mitochondrial diseases such as fatal infantile cardioencephalomyopathy, Leigh syndrome, and Charcot-Marie-Tooth disease, a neurodegenerative disorder. These diseases have a common symptom of locomotive dysfunction. However, the mechanisms of their pathogenesis remain unknown, and no fundamental medications or therapies have been established for these diseases. In this study, we demonstrated that the glial cell-specific knockdown of *Scox* perturbs the mitochondrial morphology and function, and locomotive behavior in *Drosophila*. In addition, the morphology and function of synapses were impaired in the glial cell-specific *Scox* knockdown. Furthermore, *Scox* knockdown in ensheathing glia, one type of glial cell in *Drosophila*, resulted in larval and adult locomotive dysfunction. This study suggests that the impairment of *Scox* in glial cells in the *Drosophila* CNS mimics the pathological phenotypes observed by mutations in the *SCO2* gene in humans.

## Introduction

*Synthesis of cytochrome c oxidase 2* (*SCO2*) is a highly conserved gene in a wide variety of species across prokaryotes and eukaryotes^1^. The human *SCO2* gene encodes a mitochondrial metallochaperone that is involved in the transport of copper to the CuA site, the copper oxidoreduction center of cytochrome c, together with COX17, SCO1, and COA6, and is essential for the assembly of complex IV of the respiratory chain^2, 3^. The assembly of complex IV in humans requires 13 subunits, including SCO2, which functions as a thiol-disulfide oxidoreductase to oxidize disulfide bonds of copper-binding proteins, such as SCO1 and COX2, and activates copper coordination to these proteins^4–6^. Pathogenic mutations in *SCO2* are known to cause mitochondrial diseases such as fatal infantile cardioencephalomyopathy and Leigh syndrome^7, 8^, and Charcot-Marie-Tooth disease (CMT)^9^, a neurodegenerative disorder. Patients with these diseases are considered to develop them when both parents have heterozygous mutations and the mutations of each parent are inherited, causing loss of protein function of SCO2 resulting from compound heterozygosity with two heterozygous mutations. However, the details of how the mutations cause these diseases remain unknown.

*Drosophila* has a homolog, *Scox,* which shares 74% homology with human *SCO2*. In this study, to gain further insight into the pathogenic mechanism of the diseases caused by impaired SCO2 function, we investigated the effects of *Scox* knockdown in the central nervous system (CNS), including neuronal cells and glial cells, on the *Drosophila* locomotive behavior. Glial cell-specific *Scox* knockdown resulted in locomotive dysfunction following the perturbation of mitochondrial function. This study revealed a novel aspect of the pathogenic mechanism of these diseases.

## Results

### Glial cell-specific *Scox* knockdown reduces locomotive ability

**Figure 1:**
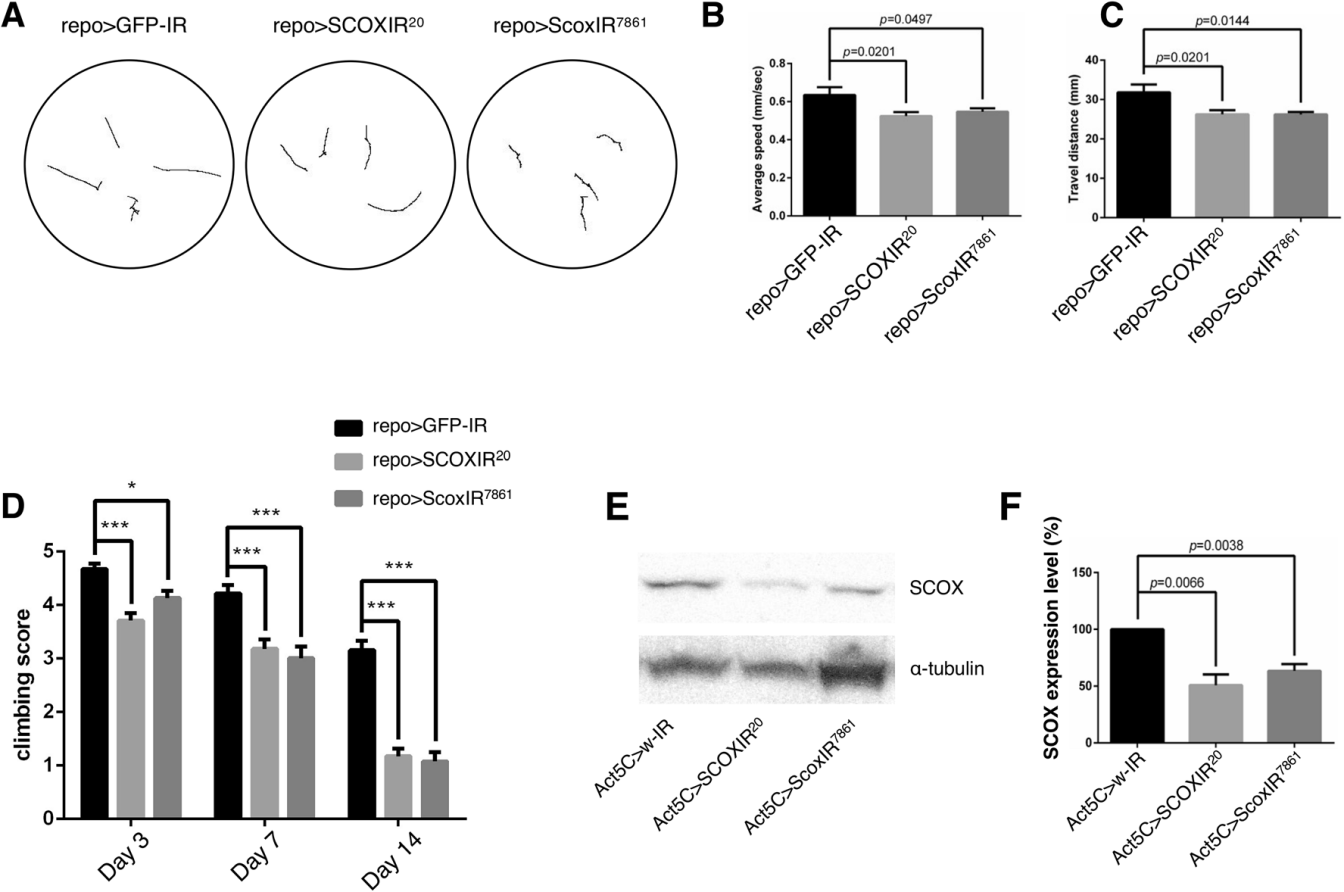
Glial cell-specific *Scox* knockdown reduces locomotive activity in larval and adult stages. The locomotive ability of larvae was tested in a crawling assay. (**A**) The crawling paths of male third instar larvae in the pan-glial cell-specific *Scox* knockdown. (**B**,**C**) Pan-glial cell-specific *Scox* knockdown reduces both the average crawling speed (**B**) and travel distance (**C**) compared with control. n = 20. (**D**) The locomotive ability of adult flies was tested in a climbing assay. Pan-glial cell-specific *Scox* knockdown reduced climbing scores compared with the control. Climbing assays were performed on days 3, 7, and 14 after eclosion, and climbing scores were calculated on each day. n = 100. *p < 0.05, ***p < 0.0001. (**E**) The expression level of SCOX was analyzed by Western blotting. Protein extracts from whole larvae were used. The expression levels of SCOX significantly decreased in both knockdown fly lines, as previously reported^11^. (**F**) Quantification of the expression levels of SCOX compared with α-tubulin. α-tubulin was used as an internal control and the relative intensities of SCOX bands normalized using α-tubulin are shown by each bar. In the *SCOX* knockdown larvae, expression levels of SCOX decreased compared with the control. n = 4. repo > GFP-IR (*UAS-GFP-IR/*+*; repo-GAL4/*+), repo > SCOXIR^20^ (*UAS-SCOXIR*^*20*^*/*+*;* *repo-GAL4/*+), repo > ScoxIR^7861^ (*repo-GAL4/UAS-ScoxIR*^*7861*^), Act5C > w-IR (*Act5C-GAL4/*+*;* *UAS-w-IR/*+), Act5C > SCOXIR^20^ (*Act5C-GAL4/UAS-SCOXIR*^*20*^), and Act5C > ScoxIR^7861^ (*Act5C-GAL4/*+*;* *UAS-SCOXIR*^*7861*^*/*+).

To investigate the effects of a reduction of *Scox* expression in the nervous system of *Drosophila*, we knocked down *Scox* in neuronal cells using two different GAL4 drivers, *neuronal Synaptobrevin* (*nSyb*)-GAL4 and *embryonic lethal abnormal vision* (*elav*)-GAL4. The neuron-specific knockdown of *Scox* induced no detectable defects in the locomotive ability of larvae or adult flies (Supplementary Fig. S1). Therefore, we next knocked down *Scox* in glial cells, which is the other cell type in the nervous system, under the control of the pan-glial GAL4 driver *reverse polarity* (*repo*)-GAL4. Although the number of glial cells in humans is estimated to be more than ten-times that of neurons, in *Drosophila*, the number is less than one-tenth^10^. However, glial cell-specific *Scox* knockdown reduced the locomotive ability of both larvae and adult flies (Fig. 1A–C). In particular, locomotion in adult flies progressively deteriorated each day after eclosion (Fig. 1D). The deficient locomotive behaviors were observed in two different *Scox* knockdown fly strains whose target sequences do not overlap, excluding possible off-target effects. In order to evaluate the knockdown efficiency of *Scox-IR* lines, Western blot analysis was carried out (Fig. 1E,F, Supplementary Fig. S2). As glial cells account for only about 10% of cells in the *Drosophila* CNS, it was difficult to evaluate the knockdown efficiency using extracts from the CNS. We therefore extracted proteins from whole larvae in which *Scox* was knocked down by the *Act5C*-GAL4 driver, ubiquitously expressing GAL4 in somatic cells. The expression levels of SCOX significantly decreased in both knockdown fly lines, as previously reported^11^ (Fig. 1E,F).

### *Scox* is essential for mitochondrial morphology and function in glial cells in the CNS

**Figure 2:**
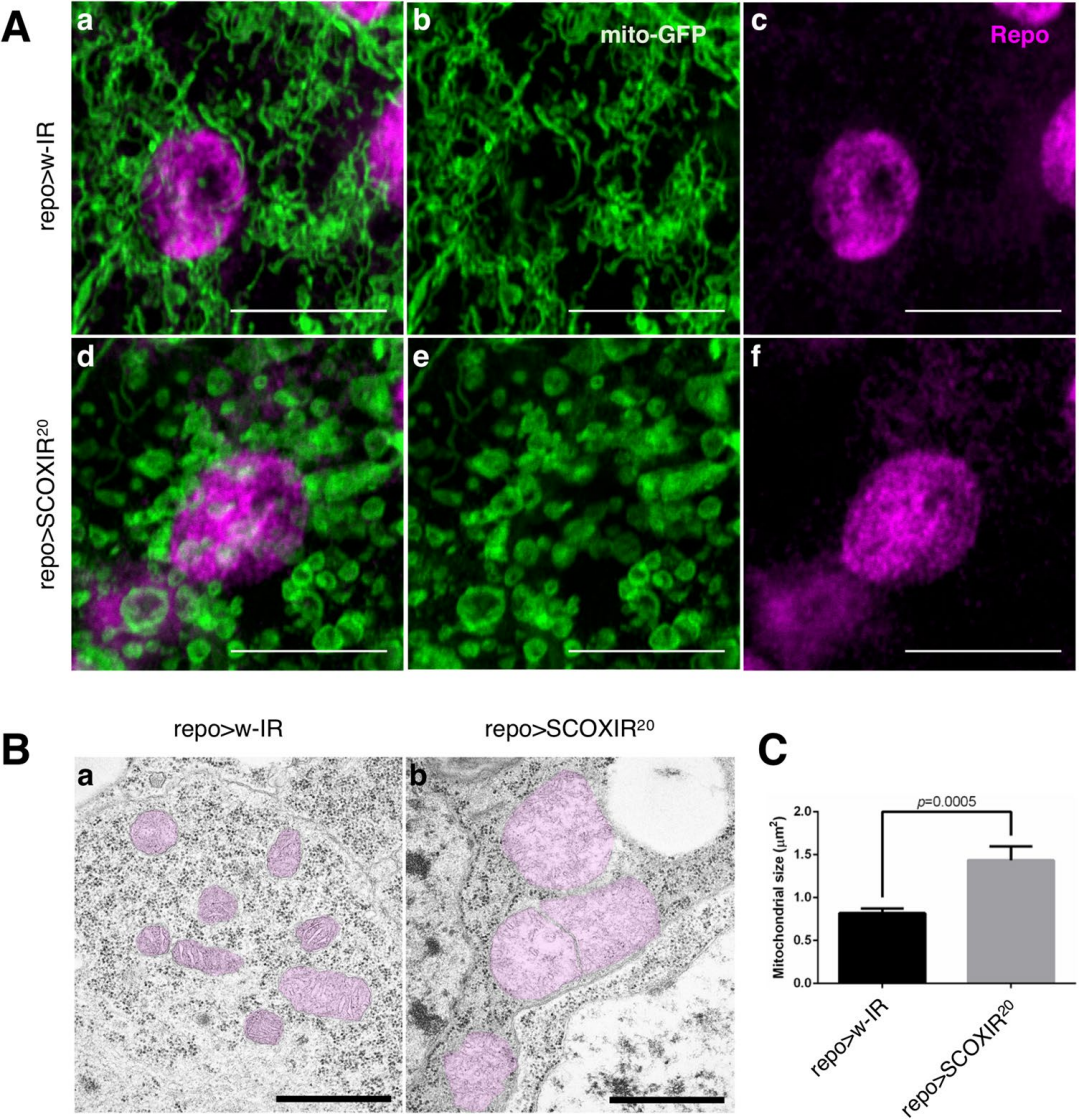
Glial cell-specific *Scox* knockdown results in morphological aberrations of mitochondria in third instar larvae. (**A**) The mitochondria in the glial cells of the CNS in third instar larvae were visualized with the mito-GFP reporter (green). The nuclei of glial cells were labeled with anti-Repo antibody (magenta). The scale bar indicates 10 µm. (a–c) *UAS-mito-GFP/+;* *repo-GAL4/w-IR*, (d–f) *UAS-mito-GFP/UAS-SCOXIR*^*20*^*; repo-GAL4/*+. (**B**) The mitochondria in the glial cells on the surface of the larval CNS were observed by transmission electron microscopy. The mitochondria are marked in pink. The scale bar indicates 1 µm. (**C**) Mitochondrial size in glial cells of the CNS was measured and quantified. *w*-IR; n = 61, SCOXIR^20^; n = 58. repo > w-IR (*repo-GAL4/UAS-w-IR*), repo > SCOXIR^20^ (*UAS-SCOXIR*^*20*^*/*+*;* *repo-GAL4/*+).

The *Scox* gene is the *Drosophila* homolog of human *SCO2* that encodes a protein required for the assembly of cytochrome c oxidase on the mitochondrial inner membrane. In mice and humans, a reduction in SCO2 leads to mitochondrial dysfunction^9, 12^. Therefore, we examined whether knockdown of *Scox* affects the morphology and function of mitochondria. Dysfunction of mitochondria-associated proteins causes fragmentation or hyperfusion of mitochondria, and negatively affects their function^13, 14^. Thus, we examined the mitochondrial morphology in glial cells of the CNS by marking them with a mitochondrial marker, mito-GFP reporter. As a result, fibrous mitochondria were observed in the control, whereas the mitochondria in *Scox* knockdown flies were rounded and fragmented (Fig. 2A). We further inspected the mitochondrial morphology in sections at 10 μm from the surface in brain lobes of the *Scox* knockdown flies using a transmission electron microscope. The size of mitochondria in this region in *Scox* knockdown flies was 1.75-times larger than that in the control (Fig. 2B,C).

**Figure 3:**
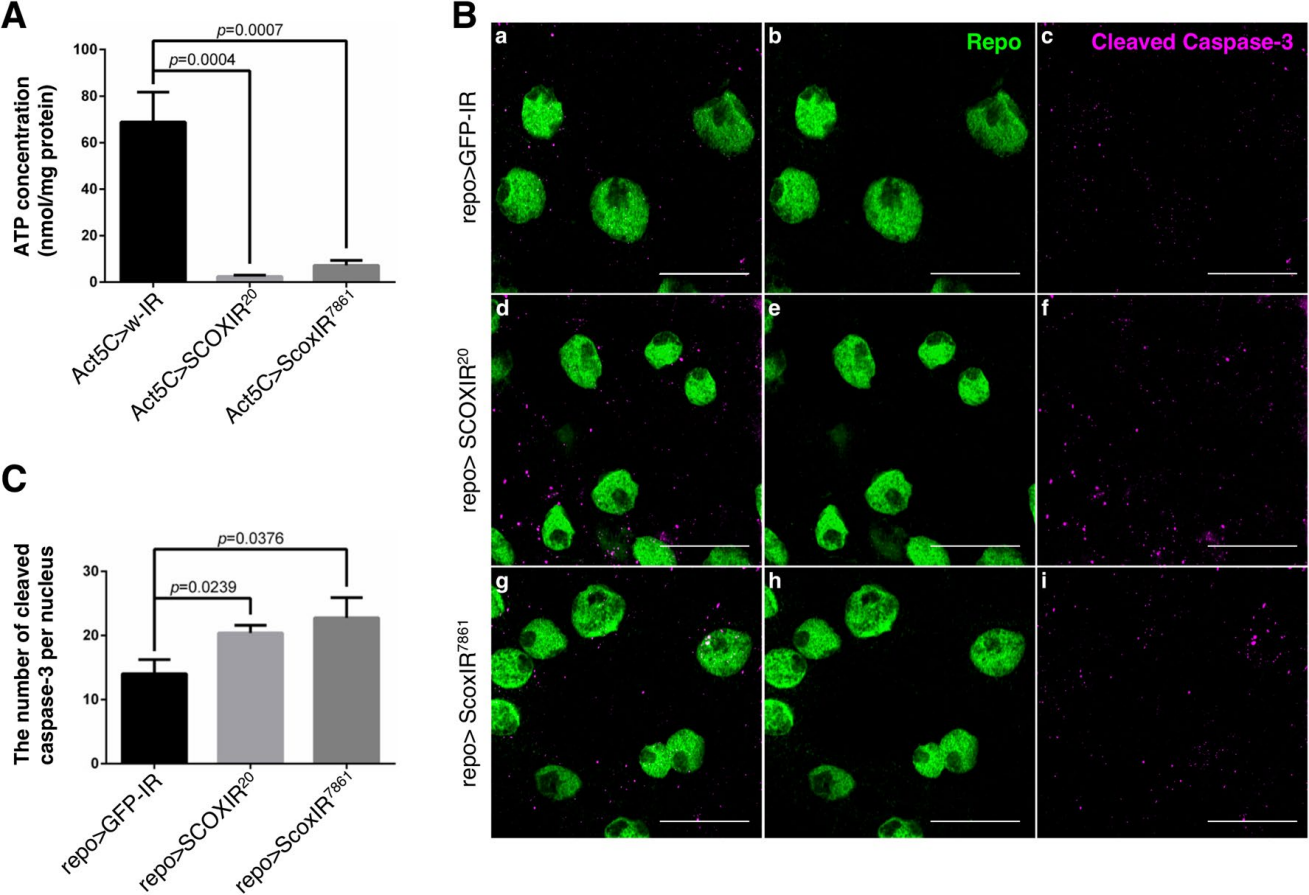
Knockdown of *Scox* induces mitochondrial dysfunction. (**A**) The amount of ATP extracted from the third instar larval CNS was measured and normalized by the amount of protein in the lysate. n = 10. (**B**) The apoptotic signals in the glial cells of the larval CNS were detected by cleaved-caspase 3 antibody (magenta). The nuclei of glial cells were labeled with anti-repo antibody (green). The scale bar indicates 10 µm. (**C**) The number of apoptotic signals in glial cells of the larval CNS were statistically analyzed. n = 10. repo > GFP-IR (*UAS-GFP-IR/*+; *repo-GAL4/*+), repo > SCOXIR^20^ (*UAS-SCOXIR*^*20*^*/*+*;* *repo-GAL4/*+), repo > ScoxIR^7861^ (*repo-GAL4/UAS-ScoxIR*^*7861*^).

As morphological abnormalities of mitochondria suggest impaired mitochondrial function, a decreased ATP level and increased apoptosis may have been induced in the *Scox* knockdown flies. The mitochondrial respiratory chain complex plays an essential role in ATP production in most eukaryotic cells. Therefore, we measured ATP levels in the *Scox* knockdown flies with *Act5C*-GAL4. Quantification of the ATP level extracted from the whole larvae revealed a reduction to 4–11% in *Scox* knockdown flies (Fig. 3A). Moreover, when *Scox* was knocked down by *Act5C*-GAL4, it was pupal lethal (data not shown).

As mitochondria become dysfunctional, they release cytochrome c, which binds to apoptosis protease activating factor 1 (APAF-1) and other proteins to form apoptosomes, thereby activating caspase-9 and caspase-3, and inducing apoptosis^15^. The observed mitochondrial morphological changes and marked decrease in ATP levels in the *Scox* knockdown flies suggested the induction of apoptosis. We therefore examined the expression level of active caspase-3 in the CNS of *Scox* knockdown flies by immunostaining. The number of active caspase-3 signals in glial cells in the CNS significantly increased in the *Scox* knockdown flies compared with the control, demonstrating that apoptosis was induced in the CNS (Fig. 3B,C).

Therefore, *Scox* plays an essential role in mitochondrial function in glial cells of the larval CNS in *Drosophila*.

### *Scox* knockdown in glial cells affects synaptic morphogenesis and function in the NMJ

**Figure 4:**
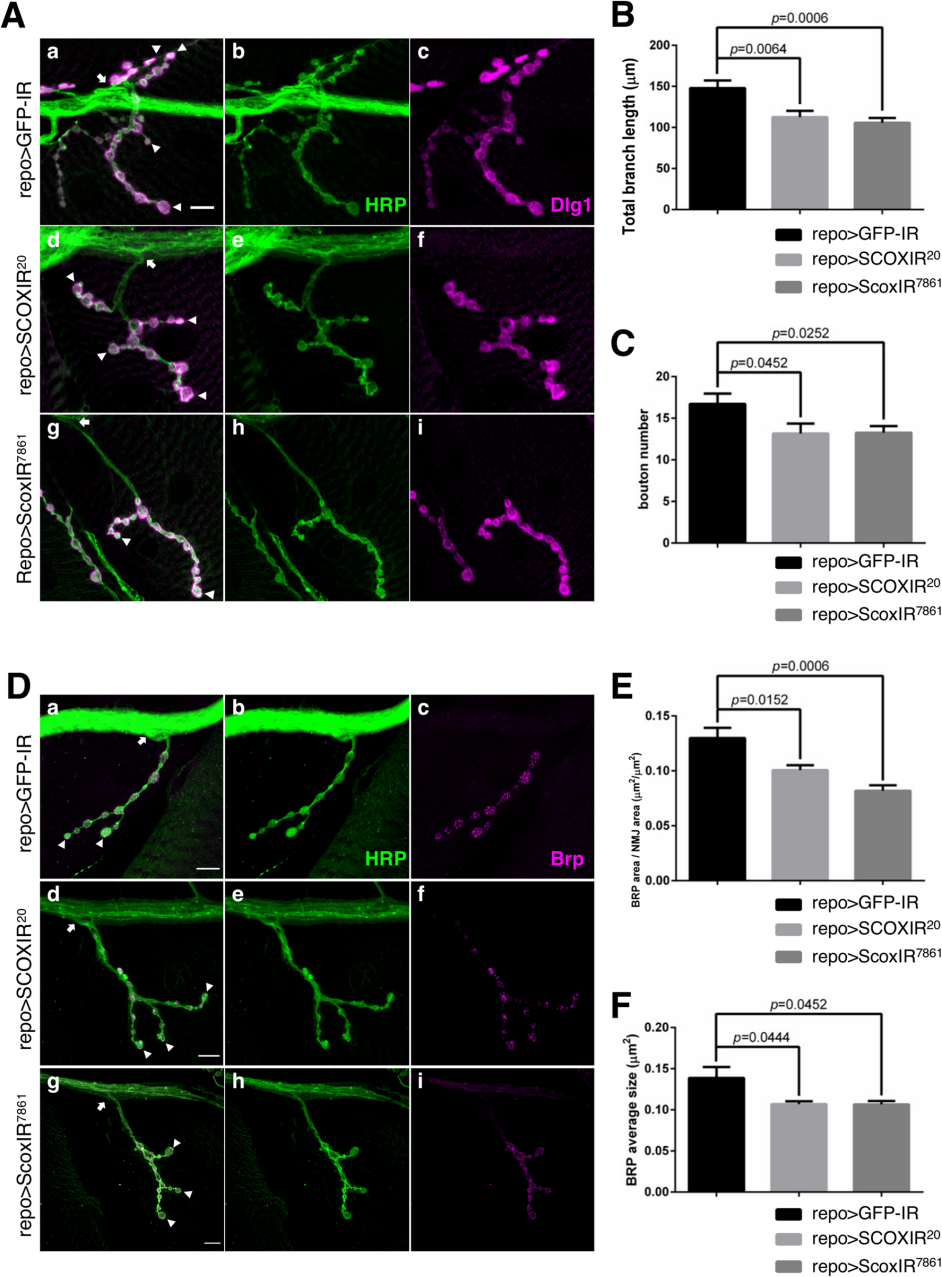
*Scox* plays an important role in the morphogenesis and function of synapses in the NMJ of third instar larvae. The synapse in the NMJ on the fourth muscle was inspected. (**A**) The pre and post synapses were marked with neuronal membrane marker anti-HRP (green) and postsynaptic marker anti-Dlg1 (magenta) antibodies, respectively. The total length of synapses between the arrow and arrowhead was measured as the total branch length. The scale bar indicates 10 µm. (**B**,**C**) Quantification of synapse branch length (**B**) and the number of boutons (**C**). GFPIR; n = 17, SCOXIR^20^; n = 19, ScoxIR^7861^; n = 21. (**D**) The active zone in the NMJ of the fourth muscle was marked by anti-Brp antibody (magenta). The number and size on Brp signals between the arrow and arrowhead were analyzed. The scale bar indicates 10 µm. (**E**,**F**) Quantification of active zone density (**E**) and active zone size (**F**). GFPIR; n = 9, SCOXIR^20^; n = 7, ScoxIR^7861^; n = 8. repo > GFP-IR (*UAS-GFP-IR/*+*;* r*epo-GAL4/*+), repo > SCOXIR^20^ (*UAS-SCOXIR*^*20*^*/*+*;* *repo-GAL4/*+), repo > ScoxIR^7861^ (*repo-GAL4/UAS-ScoxIR*^*7861*^).

The defective locomotive abilities exhibited by *Scox* knockdown flies may be due to functional abnormalities of motor neurons. Therefore, we examined the morphology and function of neural synaptic terminals at the neuromuscular junctions (NMJs) in larvae with glial cell-specific knockdown of *Scox* (Fig. 4). We measured the total length of synaptic branches and the number of boutons in the NMJs on the fourth muscle in larvae with glial cell-specific knockdown of *Scox*. We quantified the length of the synaptic branches of NMJs. The length of each synaptic branch was shortened in the *Scox* knockdown flies (Fig. 4A,B). Moreover, the number of boutons was significantly reduced compared with the control (*p* < 0.05) (Fig. 4A,C). On the other hand, neuron-specific knockdown of *Scox* induced a defect in bouton number, but not in synaptic branch length (Supplementary Fig. S3).

The presynaptic terminal has a special region called the active zone, where synaptic vesicles fuse to release neurotransmitters into the synaptic cleft and transmit signals to the next synapse or muscle. Therefore, we performed immunostaining with anti-Bruchpilot (Brp) IgG, a marker of the active zone, to investigate the density and size of the active zone in the NMJs of glial cell-specific *Scox* knockdown flies (Fig. 4D). As a result, the density of the active zone relative to the area of the synapse was reduced in the *Scox* knockdown flies (Fig. 4D,E) and the size of each active zone was small, suggesting reduced synaptic function in the *Scox* knockdown flies (Fig. 4F).

Glial cells have many functions, such as protecting and supplying nutrients to neurons, and their abnormalities may lead to the demyelination of neurons and defects in signal transmission^16–18^. Therefore, knockdown of *Scox* may result in deficits in mitochondrial function, which consequently induce defects in glial cell functions, such as protection and homeostasis of neurons, resulting in abnormal morphology and neuronal dysfunction.

### Knockdown of *Scox* in ensheathing glia is responsible for the locomotion deficit

**Figure 5:**
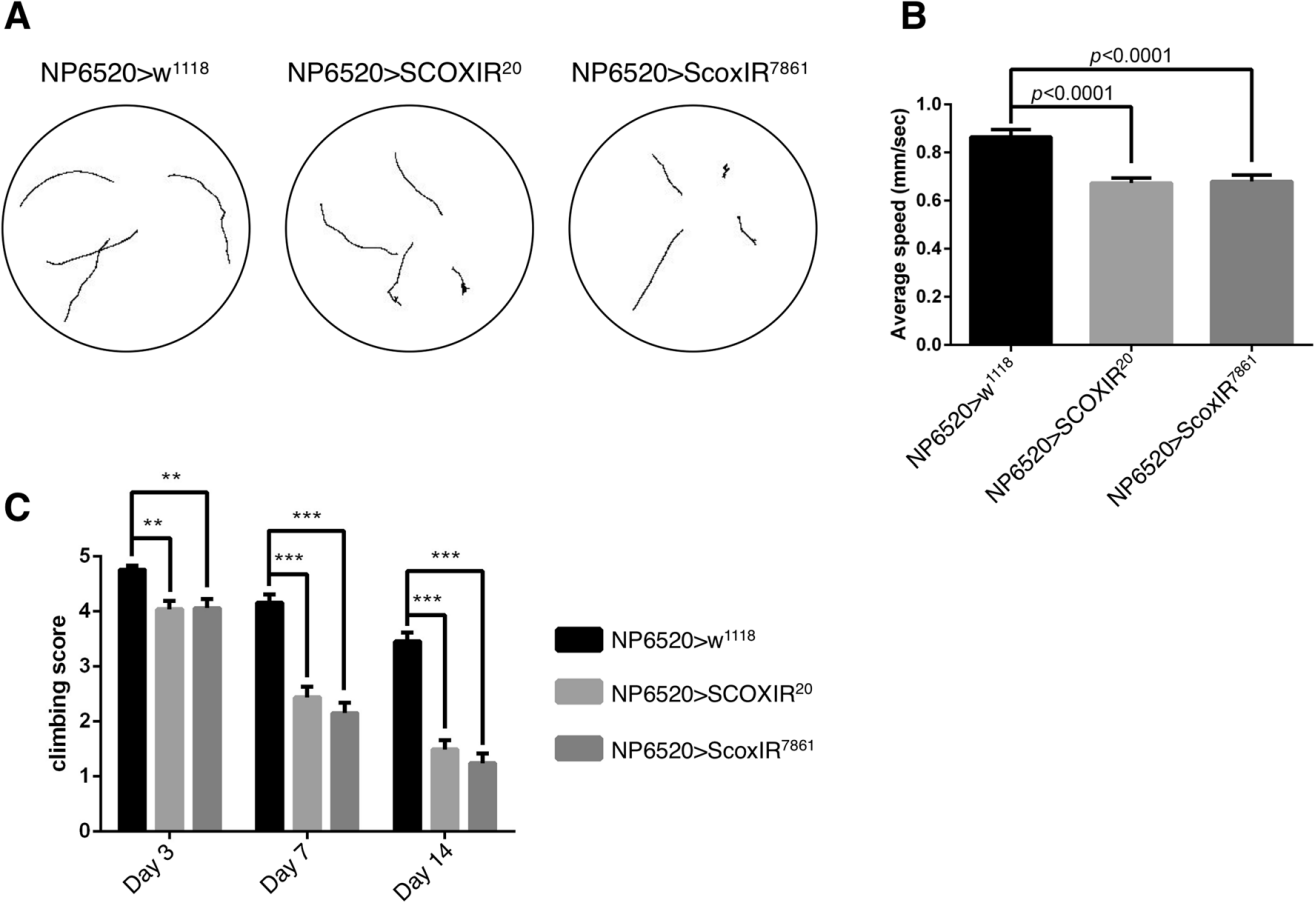
Knockdown of *Scox* in the ensheathing glia is responsible for the reduction of locomotive activity. The larval locomotive ability was examined in a crawling assay. (**A**) The crawling paths of male third instar larvae with ensheathing glia-specific *Scox* knockdown. (**B**) Ensheathing glia-specific *Scox* knockdown larvae reduced the average crawling speed. n = 20. (**C**) The locomotive ability of adult flies was measured by a climbing assay. As in the larvae, ensheathing glia specific *Scox* knockdown reduced climbing scores. Climbing assays were performed on days 3, 7, and 14 after eclosion, and climbing scores were calculated on each day. n = 100. **p < 0.01, ***p < 0.0001. NP6520 > GFP-IR (*UAS-GFP-IR/*+*;* *NP6520-GAL4/*+), NP6520 > SCOXIR^20^ (*UAS-SCOXIR*^*20*^*/*+*;* *NP6520-GAL4/*+), NP6520 > ScoxIR^7861^ (*NP6520-GAL4/UAS-ScoxIR*^*7861*^).

Mammals have four types of glial cells, whereas *Drosophila* has five types of glial cells in the CNS that function similarly to mammalian glial cells, and each of the five types of glial cells has one of the functions corresponding to the four types of mammalian glial cells or the blood–brain barrier^19–21^. *repo*-GAL4 is expressed in all glial cells in *Drosophila* and we noted decreased locomotive ability in flies in which *Scox* was knocked down by *repo*-GAL4 (Fig. 1). We investigated which of the five types of glial cells was responsible for the decreased locomotive ability in larvae and adult flies. *NP2222*-GAL4, *NP3233*-GAL4, *NP6293*-GAL4, *NP6520*-GAL4, and *Moody*-GAL4 specifically express GAL in cortex glia, astrocyte-like glia, perineurial glia, ensheathing glia, and subperineurial glia, respectively^22–25^. Among the five types of glial cells, larval crawling speed was reduced only when *Scox* was specifically knocked down in the ensheathing glia, whereas knockdown in the other types of glial cells caused no difference (Fig. 5A,B, Supplementary Fig. S4). Moreover, adult flies with ensheathing glial cell-specific *Scox* knockdown exhibited a decrease in locomotion (Fig. 5C). As in the case of knockdown of *Scox* by *repo*-GAL4, the locomotion of adult flies progressively deteriorated with each passing day.

In some neurodegenerative diseases, demyelination of the myelin sheaths of Schwann cells and oligodendrocytes is the cause of disease onset^26, 27^. As ensheathing glia most closely match oligodendrocytes in mammals, even though myelin is not formed in the *Drosophila* CNS^10^, dysfunction of *Scox* may lead to defects in locomotion because of impairment of the protection of nerve axons by glial cells.

## Discussion

The *Scox* gene is ubiquitously expressed throughout development and functions in the mitochondrial respiratory chain reaction to produce ATPs^11, 28^. The *Scox* knockdown flies by *Act5C*-GAL4, which is a ubiquitous GAL4 driver, exhibited larval locomotive defects (data not shown) and died before hatching from pupae, with a significant decrease in ATP levels. This is consistent with the previous report that the homozygote carrying null mutations in the *Scox* gene exhibiting a reduction of cytochrome c oxidase (COX) activity to 21% of wild-type, had reduced locomotive ability and died at the beginning of the second larval instar^28^. This suggests that *Scox* plays essential roles in development. However, the knockdown of *Scox* in neurons by *elav*-GAL4 or *nSyb*-GAL4 did not affect the locomotive ability. In *Drosophila*, another gene, *Surf 1*, a homolog of human *Surf1*, is involved in the COX assembly^29^. Similar to the knockdown of *Scox*, the knockdown of *Surf 1* by *Act5C*-GAL4 caused locomotive defects and death at the larval stage, whereas knockdown by *elav*-GAL4 caused no defects in locomotive ability or viability^30^. In addition, homozygous mutants of the several cytochrome c oxidase subunits, such as *tenured*, *levy*, and *cyclope*, which are homologs of COX V, VIa, and VIc, respectively, resulted in a developmental lethal phenotype^30–32^. Thus, the lethality during development based on the perturbation of the COX-related genes, at least *Scox* and *Surf 1*, may be not dependent on the defects of the functions in neurons. Alternatively, *Scox* and *Surf1* may complement each other in function or they are not expressed in neurons.

On the other hand, knockdown of *Scox* in glial cells caused mitochondrial dysfunction, which led to morphological and functional abnormalities at the NMJ, and resulted in larval and adult locomotive disability. We observed swollen mitochondria in glial cells of the *Scox* knockdown flies (Fig. 2). Defective oxidative phosphorylation led to a decrease in ATP level, causing mitochondrial dysfunction^33^, and toxic substances that inhibit oxidative phosphorylation and reduce the ATP level were reported to cause mitochondrial swelling^34^. Thus it is consistent with our study because the knockdown of *Scox*, which is an essential protein for the assembly of cytochrome c oxidase in the mitochondrial respiratory chain complex, reduced the ATP level and mitochondrial swelling led to mitochondrial dysfunction. Spinocerebellar ataxia type 1 (SCA1) is an autosomal dominant neurodegenerative disorder, a poly-glutamine disease, caused by the expression of an aberrant form of SCA1. Similar to our study, flies overexpressing the mutant form of SCA3, the *Drosophila* homolog of SCA1, in glial cells, but not in neurons, exhibited locomotive dysfunction^21, 35^. Therefore, some of the genes identified as neurological disorder-causing genes function in glial cells, but not in neurons. In *Drosophila*, there are six types of glial cells: perineurial glia, subperineurial glia, cortex glia, astrocyte-like glia, ensheathing glia, and wrapping glia^10, 19^. To clarify which type of glial cell *Scox* has functions, we knocked down of *Scox* using each glial cell-specific GAL4 driver. As a result, only ensheathing glia-specific knockdown of *Scox* affected both larval and adult locomotive ability. Ensheathing glia engulf neuropils, including axons, dendrites, and synapses, and regulate larval locomotion^36–38^. In mammals, axons are wrapped by oligodendrocytes and Schwan cells. Although myelination does not occur in *Drosophila*, ensheathing glia are suggested to closely match mammalian oligodendrocytes based on the function of wrapping axons^19^. As mutations in *SCO2*, a mammalian homolog of *Scox*, cause CMT type 4, which is a group of demyelinating peripheral neuropathy, perturbation of *SCO2* in glial cells may play an essential role in the pathogenesis of CMT. Glycolysis affects the maintenance of post-myelinated oligodendrocytes more than mitochondrial respiration, and demyelination disorders resulting from mitochondrial perturbation are linked to increased ROS and aberrant lipid metabolism, but not ATP production^39–41^; therefore, further analysis as to whether our *Drosophila* model is useful for CMT type 4 is required. Most *Drosophila* models established thus far for CMT are designed for the axonal type CMT because *Drosophila* lacks myelin sheaths and Schwan cells^42^. However, the present study suggests that the development of models mimicking some demyelinating type CMT is also possible in *Drosophila*.

In this study, we suggested that knockdown of *Scox*, the *Drosophila* homolog of human *SCO2*, which is a causal gene for CMT, in glial cells leads to locomotive disability with neuronal morphological and functional defects resulting from mitochondrial impairments. Until now, due to the lack of myelination, the importance of assessing the mechanism of demyelinating disorders in *Drosophila* has been overlooked. However, as a commonality between mammalian and *Drosophila* glia linked to ensheathing axons, such as the regulation of axon ensheathment by peripheral glia regulated by EGFR signaling^43–45^, was recently found, analyses of the glial roles in neurological dysfunction in *Drosophila* may increase.

## Methods

### Fly stocks

Fly (*Drosophila melanogaster*) strains were maintained on standard food containing 10% glucose, 5% corn flour, 4% dry yeast, 3% rice bran, and 0.65% agar at 25 °C. Fly stocks of UAS-*GFP-IR* (9331), UAS-*w-IR* (33623), UAS-*mitoGFP* (8442), *repo*-GAL4 (7415), *nSyb*-GAL4 (51635), *elav*-GAL4 (8760), and *Act5C*-GAL4 (4414) were sourced from the Bloomington Drosophila Stock Center (BDSC, IN). UAS-*ScoxIR*^*7861*^ (7861) targeting the region corresponding to amino acid positions 138 to 227 was obtained from the Vienna Drosophila Resource Center (VDRC). UAS-*SCOXIR*^*20*^ targeting the region corresponding to amino acid positions 200 to 251 was previously reported^28^. *w*^*1118*^ (108736), *NP2222*-GAL4 (112830), *NP3233*-GAL4 (113173), *NP6293*-GAL4 (105188), and *NP6520*-GAL4 (105240) were obtained from the Kyoto Stock Center (DGRC, Kyoto). *Moody*-GAL4 was gifted by Dr. Ulrike Gaul (LMU Munich, Munich, Germany). In order to minimize the genetic background effects, the fly lines used in this study were backcrossed five times with the *w*^*1118*^ strain.

### Crawling assay

Crawling assays were adapted from the previously reported method with slight modification^46^. Male third instar larvae were washed in phosphate-buffered saline (PBS) to remove food and then placed on an agar plate containing 1.7% agar. After larvae started moving, videos were taken with a digital camera for 1 min. Then, recorded videos were converted to AVI files using a MOV to AVI converter (MPEG-Streamclip), and analyzed by ImageJ (NIH, USA) with the wrMTrck plugin (developed by Dr. Jesper Søndergaard Pedersen) to track the movement of larvae and trace the path of movement.

### Climbing assay

Climbing assays were carried out in the same manner as described previously^47^. Flies were bred at 28 °C to promote the expression of GAL4 and 20 newly eclosed adult male flies were collected for experiments. Flies were put into a glass tube and tubes were tapped to collect flies at the bottom. Flies were allowed to climb the wall for 30 s and videos were filmed of them moving. This process was repeated a total of 6 times. To calculate climbing scores, the height each fly climbed was scored from 0 to 5 points at 2-cm intervals from the bottom. For each fly’s climbing score, means and standard errors were calculated, and climbing assays were performed 3, 7, and 14 days after eclosion.

### Protein isolation and Western blot analysis

To detect SCOX, proteins were extracted from 5 larvae. Larvae were heated in 0.1 M Tris–HCl (pH 7.6) and cOmplete Mini, EDTA-free (11836170001, Roche Diagnostics) for 2 min at 95 °C, and homogenized in sample buffer containing 50 mM Tris–HCl (pH 6.8), 2% sodium dodecyl sulfate (SDS), 10% glycerol, 0.1% bromophenol blue, and 1.2% β-mercaptoethanol. Samples were then heated at 95 °C for 3 min and centrifuged. The supernatants containing proteins were electrophoresed on a SDS–polyacrylamide gel containing a 12–20% gradient of acrylamide. Afterward, proteins were blotted onto polyvinylidene difluoride membranes (Bio-Rad), and blotted membranes were immersed in Tris-buffered saline containing 0.1% Tween-20 (TBST) and 5% skim milk for 1 h at 25 °C. Then, membranes were incubated with guinea pig anti-SCOX IgG (1: 1500) at 4 °C for 20 h. After incubating with anti-SCOX IgG^11^, the membranes were washed with TBST followed by incubation with horseradish peroxidase (HRP)-conjugated secondary antibody (1: 10,000, GE Healthcare) at 25 °C for 1 h. Proteins were detected using ECL select Western blotting detection reagents (GE Healthcare) and images were captured and analyzed using AE-9300H Ez-Capture MG (ATTO). The complex of the primary antibody and HRP conjugated secondary antibody was stripped from the membrane, which was incubated with anti-α-tubulin IgG (1: 10,000, 12G10, DSHB) at 25 °C for 1 h.

### Immunostaining of synapse and active zone at neuromuscular junctions (NMJs)

To visualize synapses and active zones at NMJs, male third instar larvae were dissected in PBS (calcium-free saline) and fixed in 4% paraformaldehyde (PFA) in PBS at 25 °C for 20 min. After fixation, they were washed 3 times with PBS containing 0.3% Triton X-100 (PBST) for 10 min. After incubation with PBS containing 0.15% Triton X-100 and 10% normal goat serum (NGS) for 30 min, samples were reacted with mouse anti-Dlg1 IgG (1: 200, 4F3, DSHB) or mouse anti-Bruchpilot (Brp) IgG (1: 200, nc82, DSHB) at 4 °C for 16 h. Then, samples were washed 3 times with PBST for 10 min, and anti-mouse IgG labeled with Alexa 594 (1: 200, A-11032, Molecular Probes) and FITC-conjugated goat anti-HRP IgG (1: 200, Jackson ImmunoResearch) were reacted with samples for 2.5 h. After washing 3 times with PBST for 10 min, stained samples were mounted with ProLong Diamond Antifade Mountant (P36961, Invitrogen) and observed using a confocal laser scanning microscope (A1 HD25, Nikon or LSM880 with Airyscan, ZEISS). Synapses and active zones on the fourth muscle in A2 to A6 segments were analyzed by ImageJ Fiji software (NIH).

### Immunostaining of repo and mitochondria or active caspase-3 in the brain lobe

To visualize repo and mitochondria or active caspase-3 in the CNS, male third instar larvae were dissected in PBS (calcium-free saline) and fixed in 4% PFA in PBS at 25 °C for 20 min. After fixation, samples were washed 3 times with PBST for 10 min. They were then incubated in PBS containing 0.15% Triton X-100 and 10% NGS for 30 min, and reacted with mouse anti-reversed polarity (repo) IgG (1: 200, 8D12, DSHB) and rabbit anti-GFP IgG (1: 200, 598, MBL) or rabbit anti-cleaved caspase-3 IgG (1: 200, 5A1E, Cell Signaling Technology) at 4 °C for 16 h. After the reaction, samples were fluorescently labeled with anti-mouse IgG labeled with Alexa 594 (1: 200) and anti-rabbit IgG labeled with Alexa 488 (1: 200, A-11034, Molecular Probes) or anti-mouse IgG labeled with Alexa 488 (1: 200, A-11029, Molecular Probes) and anti-rabbit IgG labeled with Alexa 546 (1: 200, A-11035, Molecular Probes). After washing 3 times with PBST for 10 min, stained samples were mounted with Vectorshield (Vector Laboratories) and observed using a confocal laser scanning microscope (Fluoview FV 10i, Olympus).

### ATP measurement

A whole larva was homogenized in 150 µl of ATP assay buffer (ab83355, Abcam), centrifuged, and the supernatant was collected. Then, 10 µl of chilled trichloroacetic acid (ab204708, Abcam) was mixed with supernatants and samples were reacted at 4 °C for 15 min. After centrifugation, 7.5 µl of neutralization solution (ab204708, Abcam) was applied to samples and samples were reacted at 4 °C for 5 min. Fifty microliters of each sample was mixed with 50 µl of Celltiter-Glo (G7570, Promega) and reacted at 25 °C for 10 min. Luminescence was detected using Lumat LB 9507 (Berthold Technologies).

### Electron microscopy

Male third instar larvae were dissected in PBS, The CNS was collected and fixed in a solution of 2% PFA and 2% glutaraldehyde in 0.1 M cacodylate buffer pH 7.4 at 4 °C for 16 h. They were then washed 3 times in 0.1 M cacodylate buffer for 30 min each and further fixed in 2% osmium tetroxide (OsO_4_) in 0.1 M cacodylate buffer at 4 °C for 2 h. After fixation, samples were dehydrated with 50% and 70% ethanol at 4 °C for 10 min. Then, samples were dehydrated with 90% ethanol at 25 °C for 10 min and dehydrated with 100% ethanol 4 times at 25 °C for 10 min. After immersing samples in propylene oxide (PO) twice for 30 min and in 70% PO resin (Quetol-812, Nissin EM) for 1 h, PO was volatilized for 16 h. Subsequently, samples were placed in 100% resin and polymerized at 60 °C for 48 h. Polymers were sliced into 70-nm ultra-thin sections with a diamond knife using an ultramicrotome (Ultracut UCT, Leica) and samples were mounted on copper grids. They were then stained with 2% uranyl acetate at 25 °C for 15 min, washed with distilled water, and stained with lead staining solution (Sigma-Aldrich) at 25 °C for 3 min. A transmission electron microscope (JEM-1400Plus, JEOL) was used to examine grids at an acceleration voltage of 100 kV and digital images (3296 × 2472 pixels) were taken with a CCD camera (EM-14830RUBY2, JEOL).

### Data analysis

In Western blot analysis, ATP measurement, mitochondrial size measurement, crawling assays, and immunostaining of NMJs (synapse and active zone) and active caspase-3, *p*-values were calculated using the unpaired two-tailed Welch’s *t*-test. In climbing assays, *p*-values were also calculated by two-way ANOVA using GraphPad Prism version 7. All graphs present the mean ± SEM.

## Supplementary Information

Supplementary Information.
