# Supplementary material for: The function of Scox in glial cells is essential for locomotive ability in Drosophila
Source: Sci Rep. 2021 Oct 27;11:21207. doi: 10.1038/s41598-021-00663-2 (PMC8551190; doi:10.1038/s41598-021-00663-2)
Supplement: Supplementary file 1 — Supplementary Information. [file 41598_2021_663_MOESM1_ESM.docx]

**Supplementary information**

**The function of *Scox* in glial cells is essential for locomotive ability in *Drosophila***

Ryosuke Kowada^1^, Atsushi Kodani^1^, Hiroyuki Ida^3^, Masamitsu Yamaguchi^3^, Im-Soon Lee^4^, Yasushi Okada^5,6^, Hideki Yoshida^1,2,*^

^1^Department of Applied Biology, and ^2^Advanced Insect Research Promotion Center, Kyoto Institute of Technology, Matsugasaki, Sakyo-ku, Kyoto 606-8585, Japan, ^3^Kansai Gakken Laboratory, Kankyo Eisei Yakuhin Co. Ltd., Seika-cho, Kyoto, 619-0237, Japan, ^4^Department of Biological Sciences, Konkuk University, Seoul, Republic of Korea, ^5^Laboratory for Cell Polarity Regulation, Center for Biosystems Dynamics Research (BDR), RIKEN, Suita, Osaka, 565-0874, Japan, ^6^Department of Physics and Universal Biology Institute (UBI), Graduate School of Science, and International Research Center for Neurointelligence (WPI-IRCN), The University of Tokyo, 7-3-1 Hongo, Bunkyo-ku, Tokyo, 113-0033, Japan

^*^Correspondence to Hideki Yoshida, PhD, Department of Applied Biology, Kyoto Institute of Technology, Matsugasaki, Sakyo-ku, Kyoto, 606-8585, Japan. Tel.: +81-75-724-7787; Fax: +81-75-724-7787; E-mail: hyoshida@kit.ac.jp

**Supplementary Fig. 1**

**
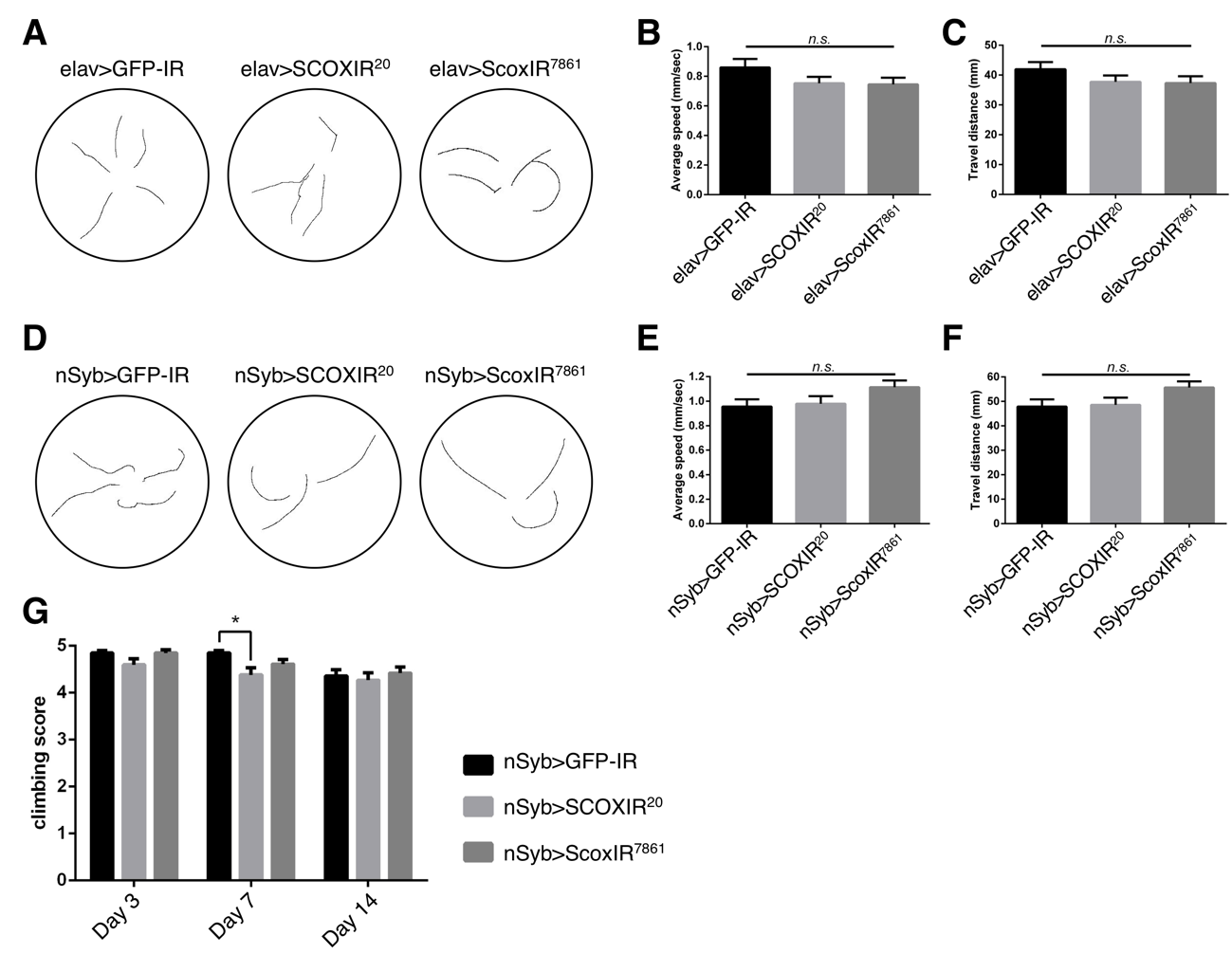
**

**Neuron-specific *Scox* knockdown does not affect locomotive ability in larval and adult stages.**

(**A**-**F**) The locomotive ability of larvae was evaluated in a crawling assay. (**A**, **D**) The crawling paths of male third instar larvae. Pan-neuron-specific *Scox* knockdown larvae exhibited no difference in average crawling speed (**B**, **E**) or travel distance (**C**, **F**). n=20. (**G**) As in the case of larvae, pan-neuron-specific knockdown of *Scox* exerted no apparent effect on climbing scores in adult flies. Climbing assays were performed on days 3, 7, and 14 after eclosion, and climbing scores were calculated on each day. n=100. *: *p*<0.05, *n.s.*: *p*>0.05. elav>GFP-IR (*UAS-GFP-IR/+; elav-GAL4/+*), elav>SCOXIR^20^ (*UAS-SCOXIR^20^/+; elav-GAL4/+*), elav>ScoxIR^7861^ (*elav-GAL4/UAS-ScoxIR^7861^*), nSyb>GFP-IR (*UAS-GFP-IR/+; nSyb-GAL4/+*), nSyb>SCOXIR^20^ (*UAS-SCOXIR^20^/+; nSyb-GAL4/+*), nSyb>ScoxIR^7861^ (*nSyb-GAL4/UAS-ScoxIR^7861^*).

**Supplementary Fig. 2**

**
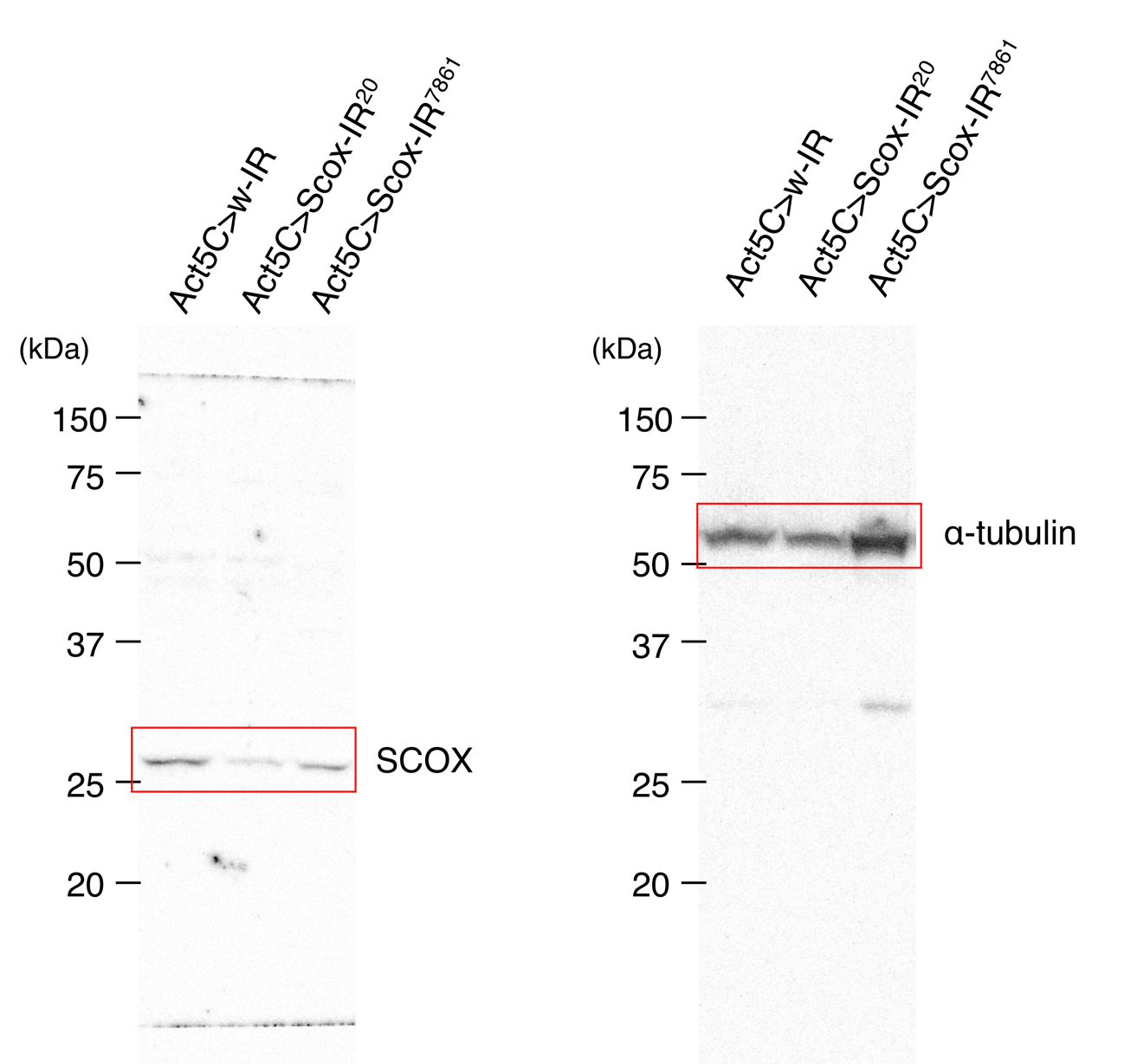
**

**The full-length blots of Figure 1E**

The full-length blots of Figure 1E. The red boxes represent the area of trimmed panels in Figure 1E. The expression level of SCOX was analyzed by Western blotting. Protein extracts from whole larvae were used. The expression levels of SCOX significantly decreased in both knockdown fly lines, as previously reported^11^.

**Supplementary Fig. 3**

**
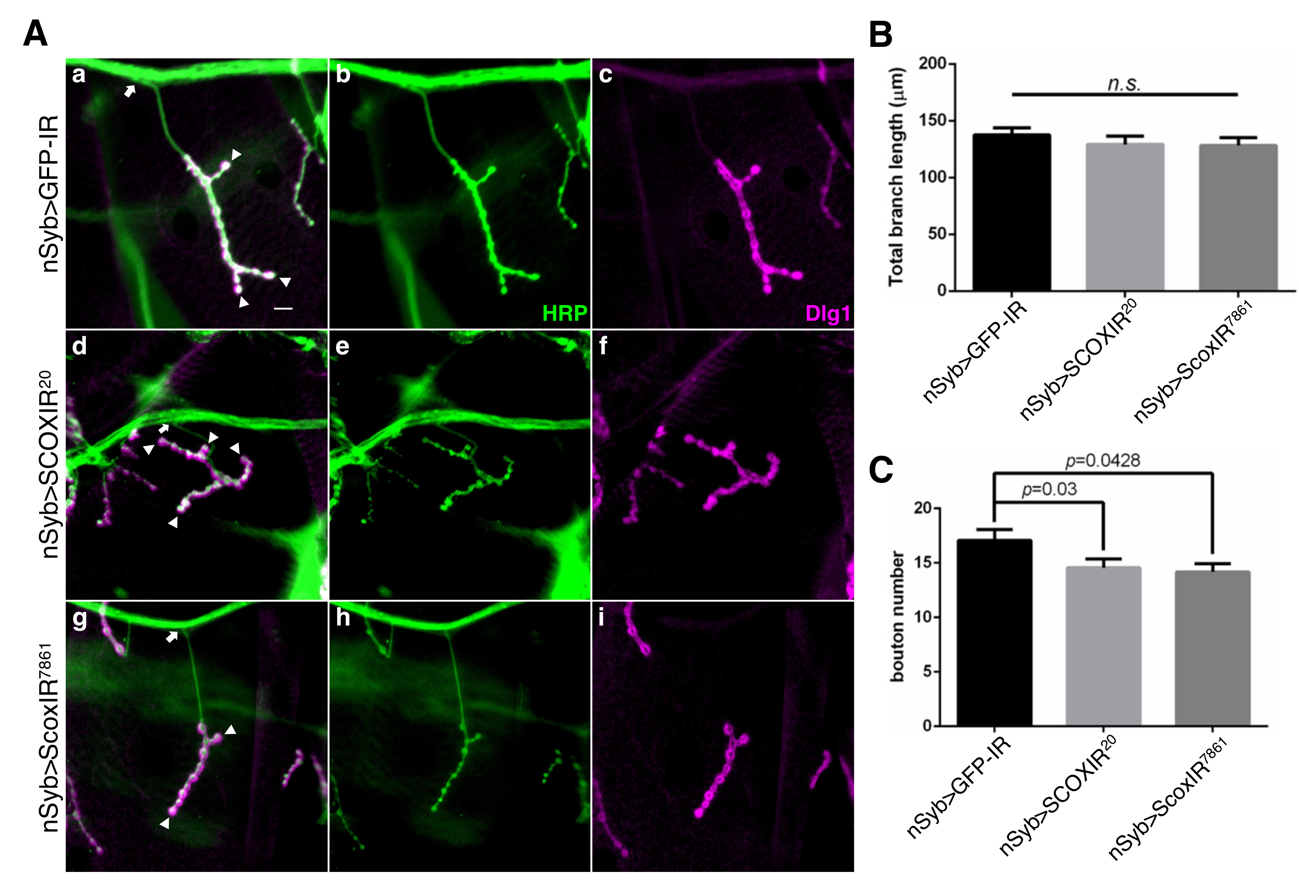
**

**Neuron-specific *Scox* knockdown exerted no apparent effect on the pre- and post-synaptic morphology in the NMJ of third instar larvae.**

The synapse in the NMJ on the fourth muscle was inspected. (**A**) The pre- and post-synapse were marked with anti-HRP (green) or anti-Dlg1 (magenta) antibodies, respectively. The total length of synapses between the arrow and arrowhead was measured as a total branch length. The scale bar indicates 10 µm. (**B**, **C**) Knockdown of *Scox* in pan-neurons exerted no effect on synapse branch length (**B**) or bouton number (**C**). *GFP-IR*; n=19, *SCOXIR^20^*; n=16, *ScoxIR^7861^*; n=21. *n.s.*: *p*>0.05. nSyb>GFP-IR (*UAS-GFP-IR/+; nSyb-GAL4/+*), nSyb>SCOXIR^20^ (*UAS-SCOXIR^20^/+; elav-GAL4/+*), nSyb>ScoxIR^7861^ (*elav-GAL4/UAS-ScoxIR^7861^*).

**Supplementary Fig. 4**

**
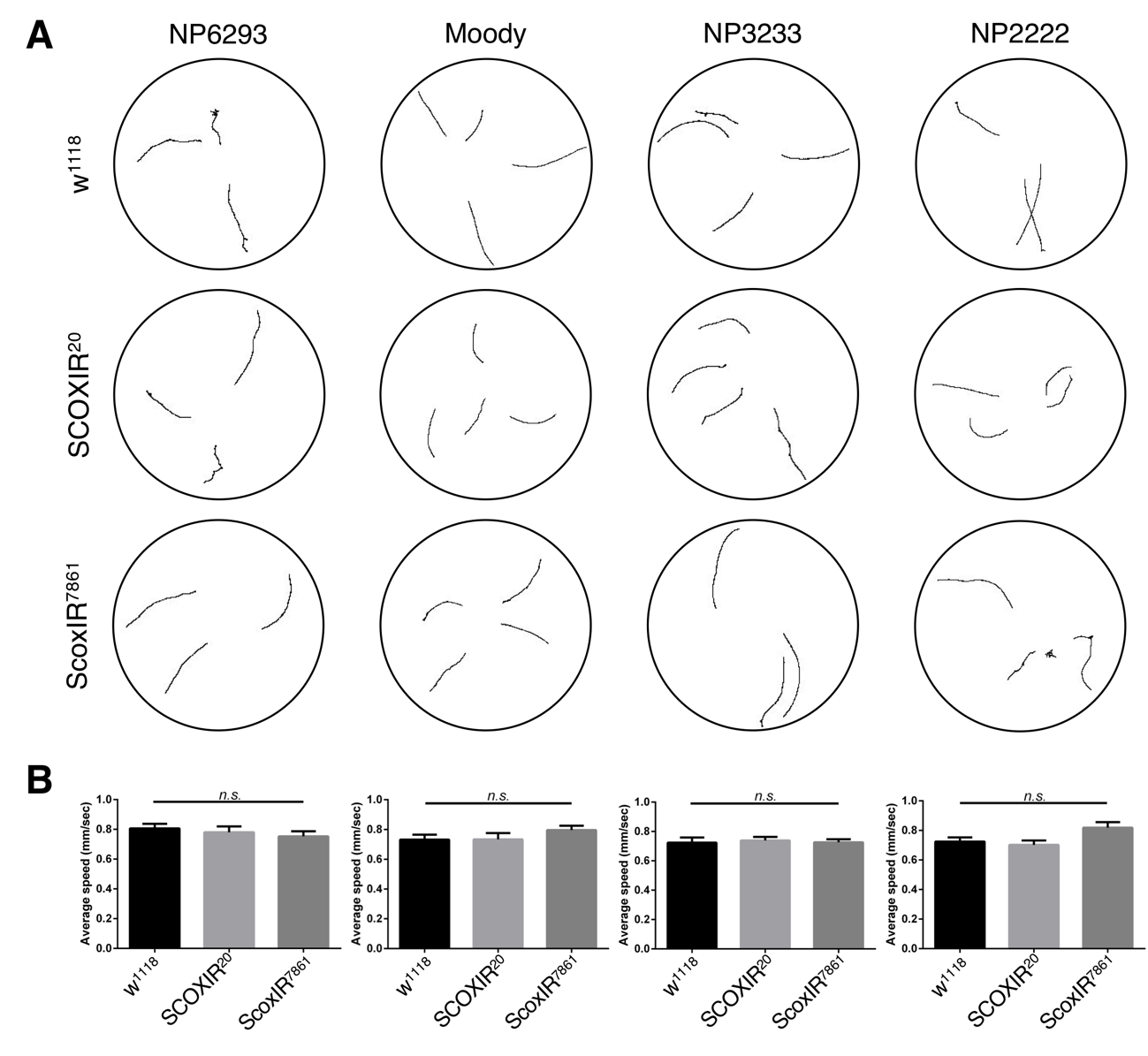
**

**Glial cell type-specific knockdown of *Scox* without ensheathing glia does not affect locomotive ability.**

The larval locomotive ability was examined in a crawling assay. (**A**) The crawling paths of male third instar larvae with cell type-specific *Scox* knockdown in each of the four types of glial cells, perineurial glia (NP6293), subperineurial glia (Moody), cortex glia (NP2222), and astrocyte-like glia (NP3233). (**B**) The average speed in each glial cell-specific *Scox* knockdown larvae did not differ. n=20. *n.s.*: *p*>0.05. NP6293>SCOXIR^20^ (*NP6293-GAL4/UAS-SCOXIR^20^*), NP6293>ScoxIR^7861^ (*NP6293-GAL4/+; UAS-ScoxIR^7861^/+*), Moody>SCOXIR^20^ (*Moody-GAL4/UAS-SCOXIR^20^*), Moody>ScoxIR^7861^ (*Moody-GAL4/+; UAS-ScoxIR^7861^/+*), NP3233>SCOXIR^20^ (*NP3233-GAL4/UAS-SCOXIR^20^*), NP3233>ScoxIR^7861^ (*NP3233-GAL4/+; UAS-ScoxIR^7861^/+*), NP2222>SCOXIR^20^ (*NP2222-GAL4/UAS-SCOXIR^20^*), NP2222>ScoxIR^7861^ (*NP2222-GAL4/+; UAS-ScoxIR^7861^/+*).
